# Important Meeting of Pharmacists

**Published:** 1920-10-23

**Authors:** 


### Important Meeting of Pharmacists.

A Meeting of the Pharmacy Unit of the Incorporated Association of Hospital Officers, the Association of Women Pharmacists, and the Public Pharmacists' Association will be held at 23 Bedford Square, W.C., on Friday, October 29, 1920. The chair will be taken at seven o'clock by Mr. F. A. Hocking, M.B.E., B.Sc. Papers will be read by Miss C. A. Andrews, Ph.C., and Mr. E. A. Andrews, Ph.C., F.C.S., on "Lord Dawson's Report and Pharmacy," to be followed by a discussion. The attendance of members (and their friends) of the above-named Societies is particularly requested.

